# The GAP project in southeastern Turkey: the potential for emergence of diseases.

**DOI:** 10.3201/eid0102.950207

**Published:** 1995

**Authors:** S Aksoy, S Ariturk, M Y Armstrong, K P Chang, Z Dörtbudak, M Gottlieb, M A Ozcel, F F Richards, K Western


### The GAP Project in Southeastern Turkey: The Potential for Emergence of Diseases

**To the Editor**: The undersigned, representing interested scientists from both Turkey and the United States, recently visited the water development projects in southeastern Anatolia, Turkey. This letter describes our observations and projections on the possible health-related consequences of these projects with specific emphasis on infectious diseases.

When new irrigation schemes are introduced into previously dry areas, disease frequently follows the new water. The Southeastern Anatolia Irrigation Project or GAP (its Turkish acronym) is one of the largest projects ever undertaken in Turkey. This water resources development program includes the construction of 22 dams and 19 hydroelectric plants on the Euphrates and Tigris rivers in southeastern Turkey. Upon completion, the project will also include an irrigation network for 1.7 million hectares of land, covering eight provinces corresponding to approximately 10% of Turkey's total population and surface area (1). In its entirety, GAP comprises investments in development projects linked to agriculture, energy, transportation, telecommunications, health care, education, and urban and rural infrastructures. To ensure the success of the project, an agency has been established (the Republic of Turkey Prime Ministry Southeastern Anatolia Project Regional Development Administration) to oversee and implement all of these projects.

The largest of the completed dams on the Euphrates River is the Ataturk Dam. It is the sixth-largest rock-filled dam in the world; its hydroelectric systems have already produced more than seven billion kilowatt hours of power since 1992 (2). Water from the Ataturk Dam reservoir is diverted to the plains of upper Mesopotamia through the Sanliurfa Irrigation Tunnel System. This system consists of two parallel tunnels, each 26.5-km long and 7.62 m in diameter, and numerous other irrigation networks and canal systems. The first water started to flow to the plains of Harran in November 1994. Additional lands will be incorporated into the irrigation scheme as the canals are completed. (The year 2020 is the target date for completion.) When fully operational, GAP is expected to double Turkey's hydroelectric production, increase irrigated areas by 50%, more than double the per capita income in the region, more than quadruple the gross national product, and create two million new jobs in the coming decade (3). The total surface area affected by the irrigation scheme is about 75,000 $km^2$; of this, 46.2% is cultivated (36% semiarid rain-fed farmland), 33.3% is dry pastures, 20.5% is forest and bush.

One of GAP's major goals is to remove the socioeconomic disparity between the country's more developed regions and the project area. For GAP to reach its targeted and sustainable economic aims, projects in various other sectors also need to be considered and integrated. In this context, the public health consequences of emerging diseases in this setting must be anticipated so that appropriate health education and disease prevention measures can be implemented.

To anticipate changing patterns in disease associated with microclimatic and other environmental changes, knowledge of existing diseases in the region is vital. Since arthropods, reservoir animals, and other intermediate hosts are involved in the transmission of many waterborne parasitic diseases, a clear understanding of the existing species—especially of insect vectors—is equally important.

Historically, occasional cases of malaria have occurred in the region; however, limited records show that this disease is clearly on the rise. Cutaneous leishmaniasis is also endemic and on the rise, but few data are available on the prevalence of the visceral form of the disease. Other common diseases in the region include bacterial and helminthic gastrointestinal infections as well as trachoma.

According to data from the Malaria Division of the Turkish Health Ministry, the reported cases of *Plasmodium vivax* malaria rose from 8,680 in 1990 to 18,676 in 1992 (4). The province of Sanliurfa (population one million in 1990), which is at the heart of the irrigated plains in GAP, has reported that malaria cases increased from 785 in 1990 to 5,125 in 1993. The numbers of cases in the first 9 months of 1994 alone were already significantly higher than those reported in 1993 (S. Aksoy, unpublished data). Although presumably *P. vivax* malaria is most common, cases of *P. falciparum* malaria have also been reported in the country. Three cases of *P. falciparum* malaria were recently documented in Izmir, which is on the Aegean Sea coast of Turkey (4). No cases of drug-resistant malaria have been reported.

Another endemic disease on the rise in the southeastern region is leishmaniasis, transmitted by biting sand flies. In Sanliurfa the number of documented cases of the cutaneous form of this disease has risen from 552 in 1990 to 1,955 in 1993. In the first 9 months of 1994 alone, the number of reported cases was more than 3,000 (S. Aksoy, unpublished data). At Sanliurfa's Diyarbakir Hospital, in 1991, in addition to cases of the cutaneous forms of the disease, there were 80 potential cases of visceral leishmaniasis (kala-azar) in children ages 2 to 10 (5). *Leishmania donovani* is often the causative agent of kala-azar, but both *L. tropica* and *L. infantum* may also be involved (6). As the economic opportunities in the GAP provinces attract populations to the region, visceral leishmaniasis may become a greater threat. The prevalence of the sand-fly species in the region, their habitats, and the future implications of the microclimatic changes for these habitats must be studied to anticipate future disease patterns.

Other prevalent pathogens in the region include *Entamoeba histolytica, Giardia lamblia, and Ascaris lumbricoides.* Of 22,468 stool samples examined in one study, over 90% carried intestinal parasites; in children from infancy to 5 years of age, 60% contained *Giardia intestinalis* (7). In a second study in Diyarbakir involving 4,670 patients (ages <1–65 years), the incidence of protozoan and helminthic infection was approximately 16% (53%, *E. histolytica*; 31%, *G. lamblia*; and 10%, *A. lumbricoides*) (8). In both studies, the incidence of amebiasis was approximately 8% to 9%. In 1989, a survey conducted among 1,001 children in four elementary schools in Sanliurfa found parasites in 88% of the stool samples examined (50% *Ascaris*, 53% *Trichuris trichiura,* 22% *Giardia*, 11% *Entamoeba coli)* (9). Ancylostomiasis, which occurs in the eastern Mediterranean, is a potential danger for the region (10).

The emergence of schistosomiasis, which can quickly reach epidemic proportions in water-related projects unless measures are taken, should not be ignored. A recent study in Sanliurfa has identified *Bulinus truncatus*, the snail vector of *Schistosoma haematobium* in the region (11). Whether other regions in GAP also harbor this species is not known, although there have been reports of these snails in the Nusaybin and Mardin regions (12). A few decades ago, sporadic cases of disease were also reported from southeastern regions (13). As microclimatic changes occur in the GAP area, the presence of these snails and the potential emergence of schistosomiasis should be closely monitored.

The costs of combating epidemic diseases can be very large, whereas the costs of prevention are much lower. Large national projects that anticipate economic benefits may sometimes overlook the distant prospects of disease. Ideally, health planning should be built into a project from its inception for small funds invested for prevalence studies early on can bring high returns later. Earlier dam projects in Senegal, Lake Volta, and Egypt have shown that unless effective measures are taken early, infections can quickly reach epidemic levels (14). The establishment of good surveillance and recording systems is an important first step.

**Serap Aksoy,* Sedat Ariturk,† Martine Y.K. Armstrong,* K.P. Chang,‡ Zeynep Dörtbudak,* Michael Gottlieb,§ M. Ali Ozcel,¶ Frank F. Richards,* and Karl Western§**
*Yale Univerity School of Medicine, New Haven, Connecticut
†Dicle University, Diyarbakir, Turkey
‡Chicago Medical School, Illinois
§National Institute of Allergy and Infectious Diseases, National Institutes of Health, Bethesda, Maryland
¶Ege University Medical Faculty, Bornova, Izmir, Turkey

### References

1. Republic of Turkey Prime Ministry. GAP action plan. Ankara, Turkey: GAP Regional Development Administration, 1993 (in Turkish).
2. Republic of Turkey Prime Ministry. GAP status report (1993). Ankara, Turkey: GAP Regional Development Administration, 1993 (in Turkish).
3. Republic of Turkey Prime Ministry. Agricultural commodities marketing survey and planning of crop pattern for GAP. Ankara, Turkey: GAP Regional Development Administration, 1992.
4. Ok UZ, Buke M, Sayiner AA, Özcel MA. Three *Falciparum* malaria cases in Izmir. Acta Parasitologica Turcica 1994;18:33.
5. Özerdem N, Mete Ö. The interpretation of diagnostic methods in kala-azar (visceral leishmaniasis) infection. Acta Parasitologica Turcica 1993;17:1-6.
6. Qy J-Q, Zhong L, Yasinzai-Mascom, M, et al. Serodiagnosis of Asian leishmaniasis with a recombinant antigen from the repetitive domain of a *Leishmania* kinesin. Trans R Soc Trop Med Hyg 1994;88:543-5.
7. Duran G, Mete Ö. The epidemiological evaluation of intestinal parasites in our region. Acta Parasitologica Turcica 1993;17:35.
8. Balikci E, Özel MF, Mete Ö. The investigation of intestinal parasites on the stool flora in patients who were admitted to microbiology laboratory. Acta Parasitologica Turcica 1993;17:27.
9. Unat EK, Akaslan I, Akaslan S, et al. Results of the parasitological examination of stools from students of our elementary schools in Sanliurfa. Acta Parasitologica Turcica 1989;13:75.
10. Unat EK, et al. Medical parasitology. Istanbul: Istanbul University Press;1983:323.
11. Sesem R, Yildirim MZ. Life cycle of *Bulinus truncatus* (Audouin 1827) (Pulmonata:Gastropoda) under laboratory conditions. Acta Parasitologica Turcica 1994;18:337.
12. Paydak F. Systematic analysis of freshwater gastropods in Diyarbakir, Urfa and Mardin. Diyarbakir Medical School Proceedings 1967;5:243.
13. Cebeci M. Intestinal diseases in East Mediterranean regions. Istanbul University Med Faculty J. 1959;22:701-5.
14. Stanley NF, Alpers MP. In. Man-made lakes and human health. London: Academic Press, 1975.

